# Proceedings of the Pathological Society of Philadelphia: Fatty Degeneration of Liver—Fistula of Gall-Bladder—Gangrene of Bladder—Encephaloid Tumor of Thigh—Primary Cancer of Pancreas—Bony Tumor of Scrotum—Cancer of Liver, and Other Abdominal Viscera—Softening of Stomach—Fungus of Testicle—Injury of Shoulder-Joint—Ovary Containing Bone

**Published:** 1858-01

**Authors:** 


### ART. XIII.—*Proceedings of the Pathological Society of Philadelphia.* Reported by the SECRETARY.

HOSPITAL BUILDING, Spruce Street, above Eighth.

THE Pathological Society of Philadelphia was organized on Wednesday evening, October 14th, 1857, by the adoption of a Constitution and election of the following officers:—

*President,* DR. GROSS.
*Vice-Presidents,* DRS. LA ROCHE AND STILLÉ.
*Treasurer,* DR. ADDINELL HEWSON.
*Secretary,* DR. DA COSTA.
*Assistant-Secretary,* DR. T. G. MORTON.

The Society then proceeded to the exhibition of specimens.

*Fatty Degeneration of the Liver.*—DR. S. W. MITCHELL exhibited a liver in a state of fatty degeneration, removed from a child, aged five and a half years, born in Georgia. The father died of phthisis, the mother is now ill with it; the boy had had repeated attacks of diarrhœa. His final illness began with vomiting and purging, easily subdued by medicine, but recurring with typhoidal symptoms after six days. A slight convulsion preceded his death by a few hours. The chief point in regard to his constitution which seemed worthy of note, was the facility with which the slightest catarrh prostrated and reduced him. This was explained after death by the presence of disease of the lung.

Post-mortem Section.—External appearance very thin. Brain healthy. Lungs; the right at the apex presented two or three small tubercular masses of the size of large peas. The intestines were generally congested, but offered no evidence of inflammation. The glands of Peyer were healthy, as well as those of the mesentery. The stomach about the greater curve was altered by chronic inflammation. The kidneys were healthy except at the lower ends of the renal tubes, whose cavities were very generally filled up by masses of yellow matter, which tinted the divided cones of a golden color. The liver was more yellow throughout than is usual, and exhibited everywhere to the microscope the evidence of an unhealthy excess of fat. On the upper surface the fat presented itself in very distinct cresentic markings, which offered a very curious contrast to the hue of the more healthy tissue about them.

In this case fatty degeneration of the liver preceded the general development of pulmonory tubercle.

Dr. Mitchell was requested to report further on the nature of the yellow matter in the uriniferous tubes.

WEDNESDAY EVENING, Oct. 28th, 1857.

The President in the chair.

DR. MITCHELL submitted the following report regarding the kidneys presented at the last meeting:—

"The kidneys are healthy as to tissue, but the lower end of most of the tubes is filled with dark masses which tint the renal cones of a golden yellow. Under the microscope these masses are observed to be rounded, and acicularly crystallized, the needle-like composite crystals radiating from the centre of each mass. The arrangement is common to crystalline masses of certain urates, rarely to phosphate and oxalate of lime, and always to carbonate of lime. To my great surprise I found these masses to be the last-named substance,—a fact as to which I was so skeptical that I examined them repeatedly, and with great care. By scraping several cones with a knife, I collected enough for micro-chemical analysis. The acicular and radiated structure came out as they dissolved in acetic acid, which they did easily, with development of bubbles of gas. After complete dissolution, the addition of a drop of oxalic acid in solution produced a white precipitate. From these reactions several times observed, as well as from their general form, I therefore conclude the masses to be carbonate of lime. The smaller crystals higher up in the tubes were yellow and transparent, and were probably uric acid, although their amount was too small to admit of even micro-chemical research. The occurrence of carbonate of lime in human urine is very rare. It can only take place in a neutral or alkaline secretion. I have notes of a case from Wilmington, Del., which I saw two years ago. The patient was a retired merchant, who suffered with dyspepsia of an irritative nature. The deposition of the carbonate was in the form of amorphous masses, and a few such crystalline radiated forms as are seen in the case before us. In another instance the urine of a hysterical woman was loaded with quantities of pulverized chalk, placed in it to deceive the attending physician."

In answer to a question whether the urine had been examined during life, Dr. Mitchell stated that it had, but that it had presented nothing remarkable.

*Fistula of the Gall-bladder.*—DR. MORTON exhibited a specimen of fistula of the gall-bladder, taken from a female aged sixty-five, who died from diarrhœa; she had been subject to attacks of jaundice, accompanied with great pain in the region of the liver for many months. In July, 1857, she noticed a tumor over the region of the liver, which continued to increase, and finally opened, in the month of August, and discharged a considerable quantity of fluid. The opening was near the twelfth rib, about four inches from the median line; the discharge continued from the fistulous orifice until her death.

*Atrophy.*—The liver was much increased in size, of a bright yellow color, and fatty. The gall-bladder was firmly attached to the walls of the abdomen; on opening it a fistulous orifice was found leading from the gall-bladder through the parietes of the abdomen, and opening at the point above mentioned. The walls of the bladder were much increased in thickness; numerous gall-stones completely blocked it up, obstructing also the cystic duct; no gall-stones were observed in the discharge which came from the fistulous opening. The body was much jaundiced.

DR. STILLÉ remarked that he was at a loss to understand why jaundice was present in the case, as only the cystic duct was closed; he did not consider the fatty degeneration as sufficient to account for it. With regard to the evidently fatty state of the liver, he wished to draw attention to the distinction between fatty deposit and fatty degeneration.

DR. KEATING alluded to several cases of fatty degeneration accompanied by jaundice.—A discussion took place about fatty degeneration and fatty deposit, as also whether a fatty state of liver might be produced by the administration of cod-liver oil.

DR. LEVICK did not understand how this could occur, as a fatty degeneration was always accompanied by a lowering of the system or part involved, which cod-liver oil would be more likely to remedy.

DR. DA COSTA remarked, that to tell whether oil-globules in the liver, deposited as they are in the secreting cells, were the result of a deposit or fatty degeneration, would be impossible. With regard to the fistula in the gall-bladder in the specimen before them, he pointed to the fact that in nearly all the reported cases of fistula of the gall-bladder, numerous gall-stones, and generally large ones, had been found, and these were probably the cause of the perforation.

DR. MITCHELL stated that the gall-stones in this case consisted mainly of cholesterine.

DR. GROSS remarked that some time since he had attended a female from whom a number of gall-stones escaped externally. The fistulous opening was five inches from the median line, on a level with the umbilicus. She recovered without any unfavorable symptoms. She had not been jaundiced, nor did she complain of pain when a gall-stone was passing.

*Gangrene of the Bladder following over-distension.*—Dr. HALL exhibited a specimen of gangrene of the bladder taken from a Frenchman aged thirty-six, a stout, heavily-built man, who was brought into the Pennsylvania Hospital at 4 o'clock, on Monday afternoon, the 31st September, in an extremely prostrated condition.

There was no note or history of the case from the physician who had been in attendance, but, in answer to a hurried inquiry, Dr. Hall was informed that the patient had not passed his water for the last thirty-six hours. An attempt to relieve him by the introduction of the catheter proved ineffectual. The following particulars were afterward ascertained:—

The man was an industrious journeyman in a perfumery factory. On Saturday night he had been seen to enter his lodging-room, and was not thought of again until Monday morning at the work-hour, when, not appearing as was his wont, the foreman went to look for him, and found him locked in, and apparently very ill. A physician who was sent for at 8 A.M. found the man complaining of pain in his lower belly. On asking him if he had passed his water lately, and receiving an answer in the affirmative, he thought no more of his bladder, but prescribed a carminative to relieve the pain complained of. When summoned again at 2 P.M., in haste, the man appeared to be partially delirious, but could answer questions when aroused. But when seen in the afternoon, there was a great change for the worse. The man was comatose, and in the same extremely prostrated state as noticed when brought to the hospital.

In a subsequent interview with his employer, Dr. Hall further stated, I learned that the man had been laboring under symptoms of stricture of the urethra for a considerable time previously.

There was nothing in his room to show that he had taken an opiate, or a poison of any kind.

At the time of his admission into the hospital, he was comatose with sighing respiration, an uncertain, frequent pulse, intermitting in its beats.

His face was angulated, his eyes sunken, the face, neck, and chest bathed with a cold sweat; his belly was exceedingly tympanitic. Local dullness well marked over the hypogastric region, extending as high as the umbilicus.

The only evidence of sensation evinced by the patient was a slight impatient grunt, or rather growl, when any pressure was made upon the distended abdomen.

A temporary improvement seemed to follow his being placed in a hot bath. His pulse went down to 85 or 90, and became more developed. The surface showed by its coloration that it responded to the heat stimulus, and we thought that he could hear when we shouted close in his ears, but he gave no other evidence of sensation save an impatient plunge or two when an electric current was passed through the præcordium.

While in the bath he passed an inconsiderable quantity of urine, tinged with blood, which caused a slight diminution of percussion-dullness over the bladder.

Another attempt by Dr. Morton and myself to introduce a catheter failed.

By persevering attempts he was made to swallow stimulants. Still the coma remained as before, and the patient gradually sank into a stupor. He died at midnight.

Autopsy ten hours after death.—Head, nothing abnormal. Lungs, healthy. Liver, kidneys, spleen, ditto. There were some slate-colored patches on the peritoneum, in the hypogastric region, but there were no traces of recent peritonitis. Bladder very much dilated, and filled with decomposed, intensely alkaline urine, mixed with blood of a brown color. The fluid gave out a most pungent ammoniacal odor, and stung my fingers quite sharply in some places where there were abrasions. From the urine was deposited a soft, mealy sediment upon the posterior wall of the bladder. There were a few flakes of lymph floating about in the fluid.

The mucous membrane of the viscus was of an intensely dark-red color, more soft and spongy in its texture than normal, and covered with numerous enlarged capillaries filled with blood, ramifying all over its surface.

In the centre of one of the walls of the viscus were several striated eschars, and around these small depressions the membrane was of a greenish gray or bronzed color; interspersed with these, and illustrating beautifully the transition-stages of inflammation, were maculæ of ecchymosed blood, poured out from the over-burdened vessels. The muscular coat of the bladder also appeared to be hypertrophied.

The primary cause of the retention of urine in this case, and the obstacle to the introduction of the catheter, was seen to be a tight stricture just at the commencement of the membranous urethra. Behind the seat of stricture the membranous portion of the canal was dilated. The immediate cause of death was a spasmodic constriction of the urethra at the part already nearly closed, produced perhaps by a debauch. This brought on the retention, and the fatal issue.

DR. STILLÉ did not think that the symptoms corresponded very closely with the lesions. They were certainly not such as might be expected in a case of gangrene of the bladder.

DR. GROSS remarked that the period of retention of urine was very short. He believed it possible that the patient may have suffered from retention prior to the time it was discovered. Lymph, he stated, effused from an inflamed bladder, might assume a greenish or dark color simply by coming in contact with the urine, and this appearance might simulate gangrene.

DR. HARTSHORNE commented on the rarity of death from distension of the bladder.

*Encephaloid Tumor of the Thigh.*—DR. WOODWARD exhibited a specimen of soft medullary carcinoma from the back of the right thigh, just above the popliteal region. A similar tumor had been removed from the same patient last June, but the morbid condition had been rapidly reproduced.

The mass was a little larger than a man's fist, soft and brain-like, and consisted almost exclusively of elongated, oval, oblong, or irregular nuclei, varying from 1-6000 to 1-2000 of an inch in diameter. Intermingled with these were a few cells containing similar nuclei, and presenting the ordinary appearances of the cancer-cells. The nuclei were thick-walled, granular, without distinguishable nucleoli, and presented no reaction with dilute acetic acid.

A moderately firm tunic of connective and elastic tissue, imbedded in which were many of the above-described nuclei, invested the tumor.

*Primary Cancer of the Pancreas.*—DR. DA COSTA called the attention of the Society to a specimen of primary cancer of the pancreas. The case had been successively under the care of Drs. Mitchell and J. F. Meigs, and it was from them ascertained that the patient, a man forty-five years of age, by occupation a servant, had commenced last spring to complain of symptoms of indigestion, which yielded, however, to a great extent, to astringents and tonics. A more troublesome symptom was jaundice. Uninfluenced by medicines, it increased until the man's eyes and skin became of a most intensely yellow color. No external swelling over the liver, or in its vicinity, was perceptible. The liver seemed somewhat enlarged, and palpation showed its lower border to be rather hard, and not as smooth as is usual. Slight pain across epigastrium. About the middle of September the man presented the following symptoms: He was deeply jaundiced, had passed a blackish-yellow urine, containing bile. Bowels somewhat constipated; stools clay-colored, not observed to be fatty. Pain across epigastrium and hypochondrium, extending to back. Slight if any symptoms of indigestion. Occasional vomiting. He had emaciated somewhat, yet by no means strikingly so. His spirits were good. No ascites or anascara. No local tumor perceptible in the abdomen.

He continued in this state until the 12th of October, when he was suddenly seized with an attack of hemorrhage from the lung, which lasted for several days. The blood was red and frothy, mixed with saliva; was spat up in considerable quantity, and mostly after efforts of coughing. The attack weakened him extremely. He died ten days after its commencement, so that the immediate cause of his death was pulmonary hemorrhage.

Post-mortem examination.—Skin and cellular tissues extremely yellow. Head not examined. Lungs healthy; the seat of the hemorrhage could not be found; the lower lobes were enlarged; some yellowish serum effused in pleural cavities, especially on the left side. Heart normal. On opening abdomen no fluid. Stomach normal; here and there its mucous coat seemed thicker than usual. Spleen of normal size. Liver enlarged and dense; a few, but only very few, small hard whitish masses scattered throughout its structure. In parts the liver-structure on the surface seemed slightly granular, as if in the earlier stages of cirrhosis. The color of the liver was green and mottled, some parts being very dark, inclosing others of a lighter hue. No blood transuded at any portion from the sections made. Microscopically examined the dark spots just alluded to consisted of irregular and large pigment masses. A few of the hepatic cells were filled with pigment granules, but not many; on the whole they were perfectly healthy, and fully capable of performing their functions. The fibrous tissue in the liver was not increased. The few hard white masses showed distinct cancerous elements. Gall-bladder fully distended with a thick inky bile, a few gall-stones; cystic duct dilated; hepatic duct and the common choledoch duct much compressed by a large irregular hard mass which extended across the median line backward, and proved to be, on closer examination, the head of the pancreas. Intestines healthy; so the mesentery. Pancreas firm and enlarged; its head and a few of the surrounding glands converted into a hard tumor nearly the size of a man's fist; its left extremity also somewhat harder than normal; in the middle the gland seemed of a lighter color. Pancreatic duct pervious; it ran through the base of the tumor and emptied itself into the duodenum.

A microscopical examination of the affected organ proved the hard mass to be a scirrhous tumor. It contained a large quantity of fibrous tissue, and distinct nucleated cells, some with several, others with only one large nucleus, of a round or oval shape. Some of these nuclei were free. The cells were like those met with in marked cancerous tumors. In the portion of the pancreas next to the diseased head a great deal of fat, in the shape of granules and oil-drops, was perceived; no structure, however, like that at its head; nor could gland-cells be discerned. At the splenic extremity of the pancreas gland-cells were observed, with only a trifling amount of fat, also fibrous tissue, but none of the cells or of the structure perceived at the diseased part. This portion was, therefore, although somewhat denser, not cancerous; the middle portion was in a state of fatty degeneration; the head cancerous.

DR. DA COSTA then spoke of the fact that cancer of the pancreas seemed most usually to select the head of the organ as its seat. A further report on this, as well as on the prominent symptoms of similar cases, was asked for by the Society.

DR. MOREHOUSE alluded to the hemorrhage in this case as interesting. He had seen several cases of internal cancer, with hemorrhage from the lungs occurring as the disease advanced, without the lungs being affected.

DR. HEWSON mentioned a case in which the appearance of malignant disease had been preceded by frequent attacks of hæmoptysis, without any physical signs of pulmonary disease being present.

DR. STILLÉ inquired particularly into the hemorrhage in the case just read. Might it not have been from the stomach?

DR. DA COSTA thought not. The blood was red, frothy, and voided after slight efforts of coughing.

*Bony Tumor of the Scrotum.*—DR. RICHARDSON exhibited for DR. KERR a bony tumor of the scrotum, taken from a Chinese. The mass closely resembled the two upper maxillary bones placed in apposition. In some portions it was not very firm, but in others as hard as any osseous tissue. A minute examination proved it to contain bone corpuscles.*

WEDNESDAY EVENING, NOV. 11th, 1857.

The President, DR. GROSS, and afterwards Vice-President STILLÉ, in the chair.

DR. KEATING submitted some heart-clots to the Society, which had been obtained from a man, aged sixty-three years, who was admitted at St. Joseph's Hospital in a decidedly typhoid state, and presented signs of consolidation of the right lung. The *post-mortem* examination showed in the right ventricle a firm whitish clot, of considerable size, involving the tricuspid valve, and quite closing up the mouth of the pulmonary artery: on the left a considerable clot existed, apparently obstructing the aortic

---

* This case will be found fully reported at page 79.

opening. Dr. K. believed the clots to have been formed before death, and suggested that they might have preceded, and tended to cause the pneumonia. He spoke of the rarity of clots on the right side of the heart.

Dr. Stillé remarked that many years ago he had seen a case of consolidation of both lungs, in which sudden shortness of breath was followed by signs of cardiac disturbance. About thirty hours after the commencement of the symptoms the patient died. A large clot was found in the right side of the heart invested by a distinct serous-like membrane, delicate but firm. He thought that the clot was formed originally by the rapid movement of the valvular apparatus of the heart through the highly fibrinous blood.

Dr. Da Costa alluded to a case, attended by a medical friend, where, after a blowing sound had existed for some time in the heart, it suddenly disappeared, and the left lung became dull on percussion. Dr. Da Costa has had an opportunity of seeing, at intervals, this case for the last three years. The physical signs of consolidated tissue still exist.

A discussion on heart-clots and the signs of their organization ensued, in which Drs. Richardson, Gross, Levick, Keating, and Woodward participated.

*Cancer of the Liver, and other Abdominal Viscera.*—Dr. Keating exhibited several cancerous organs removed from the body of a man, aged forty years, a stone-mason by trade, who was admitted into the medical ward of St. Joseph's Hospital on the 13th of October, 1857. He complained then of loss of appetite, a constant dull pain in the lower part of the back, and general debility. He had been bedridden with the above symptoms during the previous three months; had not been subjected to any special treatment, but had applied to a physician for advice on account of costiveness, and was ordered to take a blue pill every night.

Upon entering the hospital his aspect was so strikingly peculiar as to attract attention, and together with the constant pain in the back, extreme emaciation, and other nephritic symptoms, to cause suspicion to rest upon the kidneys as the probable seat of the morbid process under which his system appeared to be so rapidly wasting.

Upon inquiring into the state of his urinary organs his urine was found to be scanty, quite high colored, and decidedly acid. Though carefully and repeatedly subjected to the ordinary tests for the presence of albumen, nothing more than a mere trace could be discovered. The test by the sulphate of copper also failed to give the characteristic precipitate indicating the presence of saccharine matter. His stools, at first dark and of a watery consistence, soon became of a clay color, showing that the liver did not perform its function properly; and also at the same time a tympanitic state of the abdomen was observed.

During his course of medication, a tonic and diuretic plan of treatment were tried, until within a week prior to his death, when the diarrhœa returning, and the character of the stools exciting suspicion that there might be ulceration of the bowels, the oil of turpentine, together with the tincture of opium in emulsion, was ordered, under which treatment he appeared to be improving; but during the night of the 4th inst. he complained for the first time of severe pain in the epigastric region, became delirious, and died about 7 A.M. the next day.

Upon making a *post-mortem* examination of this interesting case there was found ulceration of the intestines, together with perforation of the duodenum. The pancreas and mesenteric glands were greatly enlarged, and appeared to be completely transformed into a hard mass. Some, when cut into, allowed a whitish fluid to transude.

The concretions also extended along either side of the spinal column, from the lumbar region even up as high as the fifth dorsal vertebra. This diseased state had also slightly involved the right kidney, while the liver presented a peculiarly mottled appearance, and was filled with large, hard nodules. A mass of fibrine was found in the right ventricle of the heart, and masses like miliary tubercle could be distinctly felt, as well as seen, by cutting into the structure of the left lung.

Examined microscopically by DR. DA COSTA.—In the liver all the white spots were cancerous; they yielded a fluid on pressure, which contained large nuclei, with nucleoli, and a considerable amount of granules and oil-drops. In the harder portions many very distinct cells, with many nuclei, as also free nuclei, and a few oil-globules were present; no fibrous structure; epithelial cells, unaltered, in intervening parts. Mesenteric glands—nuclei, and small cells, much fat, and cells filled with oil-globules, obscuring nuclei; many granules, which abounded in soft portions. Clot in heart, fibrine, granular, and small cells nothing resembling cancerous formation. Lungs not examined.

In answer to a question of Dr. Stillé, whether the glands in the intestine were healthy, Dr. Keating replied that they were.

DR. WOODWARD stated that he had examined the miliary formations in the lung, and believed them to be tubercular.

They consisted microscopically of a transparent amorphous substance, in which were imbedded a few granules and a number of shrivelled, more or less angular, and collapsed nuclei.

A discussion arose as to the coexistence of cancer and tubercle. Several cases were mentioned by different members of the Society.

*Softening of the Stomach.*—DR. GROSS exhibited a specimen of softening of the stomach by the action of the gastric juice. A boy, aged two years and a half, from whom, in June last, he extirpated the left eye on account of encephaloid, died on the 24th of October, from a relapse of that disease, in a state of great emaciation and anæmia.

Post-mortem twenty-eight hours after death.—The stomach was found to be nearly filled with weak coffee, of which the child had taken large quantities for some time past, as his almost exclusive sustenance. In handling the great cul-de-sac, the organ gave way at that point with a large rent, in consequence of the softening of its tunics from the action of its contents. The mucous membrane presented a gelatinous appearance, and the muscular fibres in the parts immediately around looked preternaturally pale, and were easily torn. The rest of the organ seemed to be healthy. Contents of stomach did not exhale any acid odor. A piece of small bowel, carefully cleaned and retained in contact by its mucous surface with the cul-de-sac of the organ for three days, did not appear to have undergone any material changes in its color or consistence.

*Fungus of the Testicle.*—DR. GROSS exhibited a fungus of the testicle, with fibroid degeneration of its tubular structure.

A. W. D., aged twenty-nine, of Montgomery County, Pa., nine years ago had a chancre and bubo, which continued two months. Four years after this he contracted another chancre on the head of the penis, which was cured in three weeks, but was followed by copper-colored scaly eruption of the integuments, by rheumatism, and nodes on the arm and tibia. Three years ago the right testicle became enlarged, hard, and painful, and four months ago a small pimple appeared on the right side of the scrotum, which gradually extended into an open sore, giving rise to a large fungus formation. This soon became the seat of an offensive, ill-looking, and profuse discharge. The organ, being almost completely degenerated, was removed a fortnight since. The wound is at present in a healing condition. There is still a slight eruption on the face, and an ulcer on the left tibia. The left testicle is also partly enlarged, but much less than the right On dissection, the affected organ was found to be almost completely deprived of its natural structure, the tubular matter being replaced by a fibroid tissue of a pale yellowish color and firm consistence. At the posterior part of the epididymis was an abscess about the volume of a small hickory-nut, occupied by a tough, yellowish, cheesy-looking substance, having a close resemblance to tubercular deposit. Dr. Gross alluded to a case of a similar kind which occurred in his practice a year ago, where the testicle had undergone the same fibroid degeneration. The man had labored for a long time under tertiary syphilis, and a large fungus existed upon the scrotum.

*Obscure Injury of the Shoulder-joint.*—DR. KEATING next exhibited a specimen of an obscure affection of the shoulder-joint. The patient, a man aged thirty-two, had died of phthisis. Eight months before his death he fell down a flight of stairs and injured his shoulder. He was treated for fracture. When first seen the arm was lengthened, and had the appearance of being dislocated.

On the head of the humerus, near its lower border, the cartilage was entirely absent in two spots. The bone at these points was hard and smooth. An irregular groove passed across the glenoid cavity of the scapula. The head of the humerus had evidently rested at these bare spots on the lower edge of this groove in the glenoid cavity.

DR. GROSS considered it a case of partial absorption of the cartilage of the head of the humerus and glenoid cavity. He did not see any evidence of fracture. It was simply a removal of the cartilage, and irregularity of the bone.

DR. HEWSON remarked that Dr. Robert Adams, of Dublin, had shown him specimens of chronic rheumatic arthritis, which closely resembled that on the table.

DR. HARTSHORNE spoke at some length about the fractures of the head of the humerus and of the glenoid cavity. He did not wish to say that this was a fracture, yet it had the appearance of one. A committee, consisting of Drs. Hartshorne, Gross, and Hewson, were appointed to examine into the exact nature of the specimen presented.

*Ovary containing Bone.*—DR. WOODWARD exhibited some pieces of bone from a large ovarian cyst. The ovary in this case contained an extensive deposit of encephaloid, and the cysts contained also hair and fat.

